# Ly9 (CD229) Antibody Targeting Depletes Marginal Zone and Germinal Center B Cells in Lymphoid Tissues and Reduces Salivary Gland Inflammation in a Mouse Model of Sjögren's Syndrome

**DOI:** 10.3389/fimmu.2018.02661

**Published:** 2018-11-16

**Authors:** Joan Puñet-Ortiz, Manuel Sáez Moya, Marta Cuenca, Eduardo Caleiras, Adriana Lazaro, Pablo Engel

**Affiliations:** ^1^Immunology Unit, Department of Biomedical Sciences, Medical School, University of Barcelona, Barcelona, Spain; ^2^Histopathology Unit, Biotechnology Program, Spanish National Cancer Centre (CNIO), Madrid, Spain; ^3^Institut d'Investigacions Biomèdiques August Pi i Sunyer, Barcelona, Spain

**Keywords:** autoimmunity, Sjögren's Syndrome, antibody targeting, SLAM family receptors, Ly9

## Abstract

Sjögren's Syndrome (SjS) is a common chronic autoimmune disease characterized by the B cell hyperactivation, lymphocyte infiltration, and tissue damage of exocrine glands. It can also present life-threatening extraglandular manifestations, such as pulmonary and hepatic involvement, renal inflammation and marginal zone (MZ) B cell lymphoma. Several biologic agents have been tested in SjS but none has shown significant efficacy. Here, we report the effects of Ly9 (CD229) antibody targeting, a cell surface molecule that belongs to the SLAM family of immunomodulatory receptors, using NOD.H-2^h4^ mice as a model of SjS-like disease. Female mice were treated with anti-Ly9 antibody or isotype control at week 24, when all mice present SjS related autoantibodies, salivary gland infiltrates, and marginal zone (MZ) B cell pool enlargement. Antibody injection depleted key lymphocyte subsets involved in SjS pathology such as MZ, B1, and germinal center B cells in spleen and draining lymph nodes without inducing a general immunosuppression. Importantly, mice receiving anti-Ly9 mAb showed a reduced lymphocyte infiltrate within salivary glands. This reduction may be, in part, explained by the down-regulation of L-selectin and alfa4/beta7 integrin induced by the anti-Ly9 antibody. Furthermore, levels of anti-nuclear autoantibodies were reduced after anti-Ly9 treatment. These data indicate that Ly9 is a potential therapeutic target for the treatment of SjS.

## Introduction

Sjögren's Syndrome (SjS) is a chronic autoimmune disease that affects the salivary and lacrimal glands with progressive dryness of mouth and eyes (1). SjS is one of the most prevalent systemic autoimmune diseases in the general population (2). It is more common in middle-aged women, with a female/male ratio of 9:1. Patients with primary SjS suffer from glandular disease alterations and develop moderate to severe systemic extraglandular manifestations that affect other organs (3). SjS patients also have increased susceptibility to suffer other autoimmune diseases such as rheumatoid arthritis or systemic lupus erythematous (4). The pathophysiology of SjS is characterized by abnormal lymphocyte infiltration around the glandular epithelial cells in the lacrimal and salivary glands. This lymphocytic infiltration can be also observed in other tissues such as the tubular epithelial cells in the kidney or respiratory epithelia (5). SjS patients present anti-nuclear autoantibodies such as anti-ribonuclearprotein Ro, and elevated levels of rheumatoid factor that correlate with clinical outcome (6, 7). Another hallmark of SjS is the B cell hyperactivity characterized by the clonal expansion of marginal zone (MZ) B cells. Increased numbers of MZ B cells, in spleen and salivary glands, have been observed in several mouse models of SjS (8). B cell hyperactivity, due to ongoing antigenic stimulation, is initially polyclonal but can progress to monoclonal B cell lymphoproliferation ultimately leading to B cell lymphoma development. Accordingly, it is well known that SjS patients have a significantly higher risk of developing lymphoproliferative diseases, being the most common MZ B-cell lymphoma, which is the most detrimental clinical complication of this disease (9, 10). The current treatments aim to reduce symptoms of the exocrinopathy as well as to control the extraglandular features of the disease. However, at this stage, no disease-modifying drugs have been shown to be effective for the treatment of SjS (11).

Ly9 (CD229, SLAMF3) is a member of the SLAM family of cell surface receptors (12). It is present on all B and T lymphocytes with very high expression levels on NKT cells and MZ B cells (13, 14). Ly9 has four Ig-like domains and a long cytoplasmic tail containing two ITIM motifs, which are docking sites for the adapter molecule SAP and tyrosine phosphatases such as SHP2 or SHIP-1 (15, 16). It functions as a homophilic adhesion molecule and a signaling inhibitory receptor (17). Moreover, aged Ly9-deficient mice spontaneously develop features of systemic autoimmunity, such as splenomegaly and the production of anti-nuclear, anti-dsDNA, and anti-nucleosome autoantibodies, indicating that the Ly9 cell surface receptor is involved in the maintenance of immune cell tolerance (18). Recently, we have shown that antibody-mediated targeting of Ly9 down-regulates B cell responses and selectively depletes splenic MZ B and B1 cells (19). Ly9 antibody targeting induces a decrease in the expression of the CD19 complex and down modulates the expression of several adhesion molecules such as L-selectin (CD62L) and alfa4/beta7 integrin (19). Here, we explore the therapeutic potential of Ly9 antibody targeting in the non-diabetic NOD.H-2^h4^ mice, a mouse model of spontaneous autoimmunity mimicking SjS (20).

Our results show that Ly9 antibody targeting selectively depletes MZ, B1 and germinal center (GC) B cells in lymphoid tissues, and decreases lymphocyte infiltration in the salivary glands and kidney by modulating B cell activation and trafficking of T and B cells to inflammation sites.

## Materials and methods

### Mice

NOD.H-2^h4^ (NOD.Cg-H2h4/DilTacUmm) mice were purchased from Jackson Laboratory and breed under specific pathogen free conditions at the animal house facility from Faculty of Medicine, University of Barcelona. 12 and 24-weeks-old female mice were used for the experiments. All 24-weeks-old NOD.H-2^h4^ female mice at this age are known to have salivary gland infiltrates and autoantibodies such as anti-Ro and anti-dsDNA (21). Mice experiments were performed according to the European Community Directive 2010/63/EU and Spanish legislation (Real Decreto 53/2013, BOE-A-2013-101337) regulating the protection and usage of laboratory animals. Experimental procedures were approved by the Ethics Committee for Animal Experiments (CEEA) of the University of Barcelona.

### *In vivo* treatment with Anti-Ly9 mAb

Two treatment approaches were assessed; a therapeutic and a preventive. For the therapeutic approach, 24-weeks-old female NOD.H-2^h4^ mice were injected with two i.p. doses of 250 μg of endotoxin-free Ly9.7.144 (IgG1) mAb or isotype control (IgG1) in sterile PBS. Ly9.7.144 mAb was generated in our lab (22). The two doses were separated by 3 weeks, since we had previously observed that a single dose of 250 μg was able to maintain its biological effect for a period of at least 26 days (19). After the 6-week treatment period, mice were euthanized and plasma and organs were collected. For the preventive approach, 12-week-old female NOD.H-2^h4^ mice a single i.p. dose of 250 μg of Ly9.7.144 mAb or isotype control for 2 weeks was given. At 14 weeks mice were euthanized for plasma and organs collection.

### Cell isolation

Splenocytes and lymph nodes cell suspensions were obtained by manual disaggregation and then treated with red blood cell lysis buffer (0.15M NH4Cl, 0.01M Tris HCL), washed and incubated in 20% heat-inactivated rabbit serum before being stained with fluorophore-labeled antibodies. Cell counts were determined by using PerfectCount^TM^ microspheres (Cytognos). Salivary Gland cell suspensions were obtained by gently chopping the organ and incubating it in RPMI 3% FBS with 0.0625 mg/ml of collagenase (Sigma) for 30 min at 37°C. Digestion was stopped by adding RPMI 5 mM EDTA. Then samples were filtered through a 70 μm cell strainer (Biologixs) and processed as described above. Bone marrow cell suspensions were obtained by perfusion of femur with complete RPMI using insulin syringes and processed as splenocytes mentioned above.

### Flow cytometry

Cell suspensions from spleen, lymph nodes, and salivary glands were incubated with the fluorophore-labeled antibodies for 45 min on ice. For intracellular labeling cells were first labeled with surface antibodies and then fixed/permeabilized with the Foxp3 staining buffer set (eBioscience) and finally stained with antibodies against intracellular antigen. The anti-mouse monoclonal antibodies B220 (RA3-6B2), CD19 (6D5), CD5 (53-7.3), CD138 (281-2), CD3 (145-2C11), CD4 (GK1.5), CD8 (53-6.7), CD3 (17A2), Ly9 (Ly9ab3), integrin beta 7 (FIB504), and CD45 (30-F11) were purchased from BioLegend; GL7 (GL-7), T-Bet (eBio4B10), PLZF (Mags.21F7), CD62L (MEL-14) and CD93 (AA4.1) were from eBioscience; CD23 (B3B4), CD95 (Jo2), RORγT (Q31-378), CD44 (IM7), and CD45RB (16A) were from BD Bioscience; CD49d (R1-2) was from Milteny Biotech; and goat anti-mouse IgM polyclonal antibody from Southern Biotech. Finally, PBS57-loaded mCD1d tetramer was kindly provided by the NIH Tetramer Core Facility. Data were acquired with LSRII Fortessa or FACSCanto II flow cytometers (BD Biosciences) and analyzed with FlowJo vX.0.7 (Tree Star, Inc) software. Flow cytometry experiments were performed as described (23).

### Ly9 receptor occupancy

Antibody occupancy of Ly9 receptor was performed by a flow cytometric assay with mAb Ly9ab3-APC (14) from BioLegend. This mAb recognizes the same epitope as Ly9.7.144. Thus, Ly9 receptor cell membrane occupancy by Ly9.7.144 mAb blocks the binding of Ly9ab3-APC (Supplemental Figure 1). In order to guarantee the persistence of the biological effects of the mAb treatment, mice that had <50% of Ly9 receptor occupancy, at the endpoint, were excluded from the study.

### Anti-nuclear autoantibody (ANA) detection by immunofluorescence

Briefly, Hep-2 cells were fixed with PBS 4% formaldehyde and permeabilized with PBS 0.05% Triton. Then, cells were blocked with PBS 5% FBS 20% rabbit serum and incubated with serial dilutions of the sera starting at 1:40 dilution. To detect IgG ANAs an anti-mouse IgG Fc FITC antibody (Jackson Immunoresearch) was used. Finally, slides were visualized on a fluorescence NIKON e600 microscope and titers were determined by two different investigators.

### Autoantibody detection by ELISAs

Briefly, high binding plates (Costar) were coated with the following antigens diluted in PBS: 3 μg/ml of IgG to detect IgM rheumatoid factor antibodies; 3 μg/ml of anti-mouse IgG (Sigma M2650) to detect total IgG; 3 μg/mL of F(ab')^2^ fragments anti-mouse IgM to detect total IgM; 10 μg/ml of calf thymus dsDNA (Sigma) to detect anti-dsDNA IgG antibodies and 10 μg/ml of Ro52 (Sigma) antigen to detect anti-Ro52 antibodies. Purified antibody anti-dsDNA (Clone HspS22, Immunotools) was used as standard. Samples were diluted 1/100 for anti-dsDNA, 1/40 for rheumatoid factor, 1/40000 for total IgG, 1/5000 for total IgM and 1/20 for anti-Ro52 followed by the next secondary antibodies: Horseradish peroxidase (HRP) conjugated goat anti-mouse IgG (Sigma; dilution 1/2000) for anti-dsDNA, total IgG and anti-Ro52; Biotin rat anti-mouse IgM (BD Pharmingen; dilution 1/2000) and HRP-conjugated streptavidin (Roche; dilution 1/5000) for IgM rheumatoid factor and total IgM. Plates were developed with TMB substrate (BD Bioscience) and read with an Epoch plate reader at 450–570 nm.

### Histology and immunohistochemistry

Mice were euthanized at indicated time-points, and salivary glands and kidneys were immediately collected. Organs were fixed in PBS 4% formaldehyde overnight. After fixation tissues were embedded in paraffin and 5 μm sections were obtained. Tissue sections were then immersed in xylene and dehydrated in ethanol. Staining was performed with hematoxylin and eosin (H&E) following standard protocols. Sections were visualized at 10 × magnifications using Nikon ECPLISE Ti-S microscope. Areas were manually localized and contoured as well as automatically calculated by using NIS-Elements software. Kidney H&E sections were assessed, blindly, by a veterinary pathologist.

Fixed paraffin-embedded salivary gland tissue sections were immersed in xylene and dehydrated in ethanol. After antigen retrieval, tissue sections were blocked with PBS 5% FBS and then primary antibodies were incubated at 4°C overnight. The mAbs rabbit anti-CD3 (D4V8L; dilution 1/100), CD4 (D7D2Z; dilution 1/100) and CD8 (D4W2Z; dilution 1/400) were purchased from Cell Signaling; rat anti-B220 (RA3-6B2; dilution 1/100) was from BD Bioscience. After the incubation of primary antibodies, the endogenous biotin was blocked with the Avidin/Biotin blocking kit SP-2001 (VectorLabs) following manufacturer's protocol and secondary antibodies were applied: anti-rabbit-biotin (dilution 1/200) and anti-rat FITC (dilution 1/100; Jackson Immunoresearch) were used to detect antigen binding. Streptavidin-A555 (dilution 1/2000) was purchased from Roche. Finally, samples were mounted with mounting media (PBS 10%Glycerol). Samples were visualized at 4, 10 and 20 × magnifications using Nikon e600 microscope.

### Splenocyte migration cell adoptive transfer experiment

Thirty-week-old NOD.H-2^h4^ mice were treated with either Ly9.7.144 or isotype control antibodies for 24 h. Isolated splenocytes from anti-Ly9 treated mice were labeled with 5 μM CFSE (Molecular Probes), whereas isotype control mice were labeled with 20 μM CMAC (Molecular probes). After staining, cells were mixed together and a total of 20 × 10^6^ cells (initial ratio of 1:1 of CFSE/CMAC cells) were i.v. administrated into an age-matched NOD.H-2^h4^ mice. Finally, 24 h after transferring the cells, mice were euthanized and spleen, lymph nodes (mesenteric, inguinal and submandibular) and salivary glands were removed and processed, as described in sections above, and the ratio between CFSE/CMAC positive cells within each organ was established.

### Statistical analysis

Differences between groups means or individual values were assessed either by unpaired or paired two-tailed Student's *t*-tests. Two-tailed *p*-values <0.05 were considered statistically significant. All statistical analyses were performed using GraphPad Prism software version 6.01.

## Results

### Selective depletion of splenic MZ, B1, and GC B cells after administration of Anti-Ly9 mAb in mice with Sjögren's syndrome

**Figure 1:**
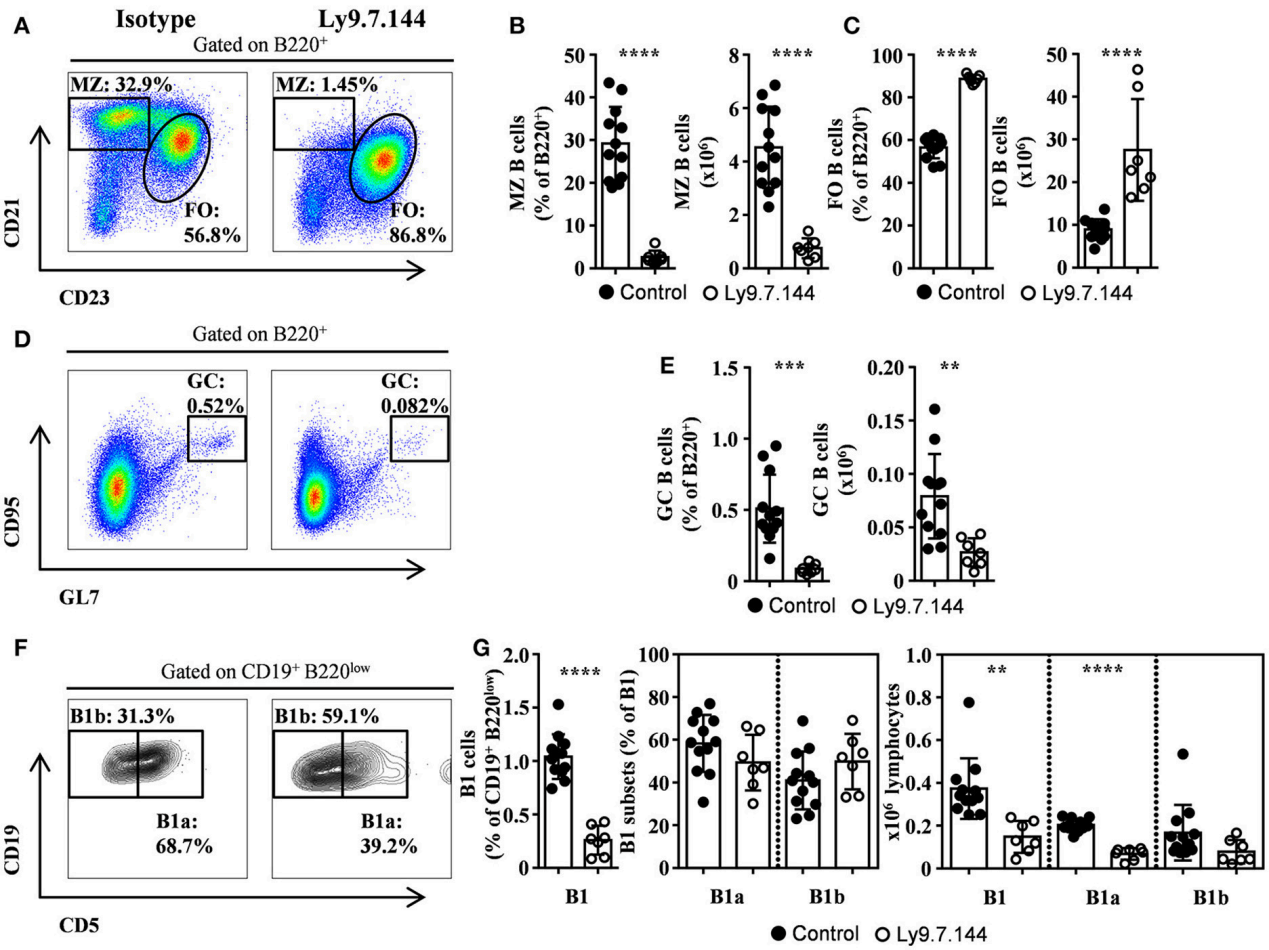
MZ, B1, and GC B cells are depleted by Ly9.7.144 administration in the spleen of SjS mice. Representative dot plots are shown for splenic MZ and follicular B cells **(A)**, GC B cells **(D)**, and B1 B cell subsets **(F)** from female NOD.H-2^h4^ mice treated with Ly9.7.144 (right plots) or isotype control (left plots). Percentage and absolute counts for the different cell subsets are shown: MZ B cells **(B)**; follicular B cells **(C)**; GC B cells **(E)** and B1 B cell subsets **(G)**. Mice treated with Ly9.7.144 mAb are represented as empty dots and mice treated with isotype control are represented as black dots. Results are expressed as mean ± SD. **p < 0.01; ***p < 0.001; ****p < 0.0001 (unpaired two-tailed *t*-test). Data are pooled from three independent experiments.

Twenty-four-week-old NOD.H-2^h4^ female mice, which spontaneously develop a SjS-like disease, were injected with agonistic anti-Ly9 mAb Ly9.7.144 (19). After 6-week treatment, mice were euthanized and lymphocyte subsets from several lymphoid organs were analyzed by flow cytometry. The injection of Ly9.7.144 mAb dramatically decreased the percentage and absolute numbers of MZ B cells in the spleen (Figures 1A,B). Concomitantly, an increment in the number of follicular B cells was observed (Figure 1C). The total number of B cells was increased (15.7 ± 1.06 × 10^6^ vs. 31.1 ± 5.07 × 10^6^; *p* = 0.0014; Isotype vs. anti-Ly9-treated mice, respectively).

Interestingly, the treatment with Ly9.7.144 induced a significant reduction of GC B cells (Figures 1D,E). Moreover, B1 B cells were also reduced, by a 50%, in percentage and numbers (Figures 1F,G), primarily due to the decrease in the number of B1a cells. This data shows that Ly9.7.144 treatment is able to target key subsets of splenic B cells strongly related to SjS pathology development and maintenance. Note that the total number of B cells before treatment were lower than those previously reported (24) but identical to those found by others (25), probably due to difference in cell counting techniques.

### The splenic T cell compartment of NOD.H-2^h4^ is modestly disturbed by Ly9.7.144 injection

A significant reduction was observed in percentage of splenic naïve CD8^+^ T cells but this reduction was not reflected in absolute numbers (Supplemental Figures 2A,B) since the total number of T cells was augmented (15.82 ± 1.1 × 10^6^ vs. 23.45 ± 1.9 × 10^6^; *p* = 0.0016; Isotype vs. anti-Ly9-treated mice, respectively). This increase was due to an increase of both memory CD4^+^ and CD8^+^cells (Supplemental Figures 2A,B). The percentages of CD3^+^ T cells were not affected by anti-Ly9 treatment (data not shown).

The effect of Ly9.7.144 injection on iNKT cells was also analyzed. We observed a reduction in percentage of total iNKT cells but not in absolute numbers (Supplemental Figures 2C,D). When we analyzed the specific subsets iNKT1, iNKT2 and iNKT17, we could not appreciate any effect of Ly9.7.144 on their percentages or absolute numbers (Supplemental Figures 2E,F). These results indicate that anti-Ly9 antibody administration had a rather mild effect on T cell subsets.

### MZ B and naïve T cells within submandibular draining lymph nodes are reduced by Ly9.7.144 injection

**Figure 2:**
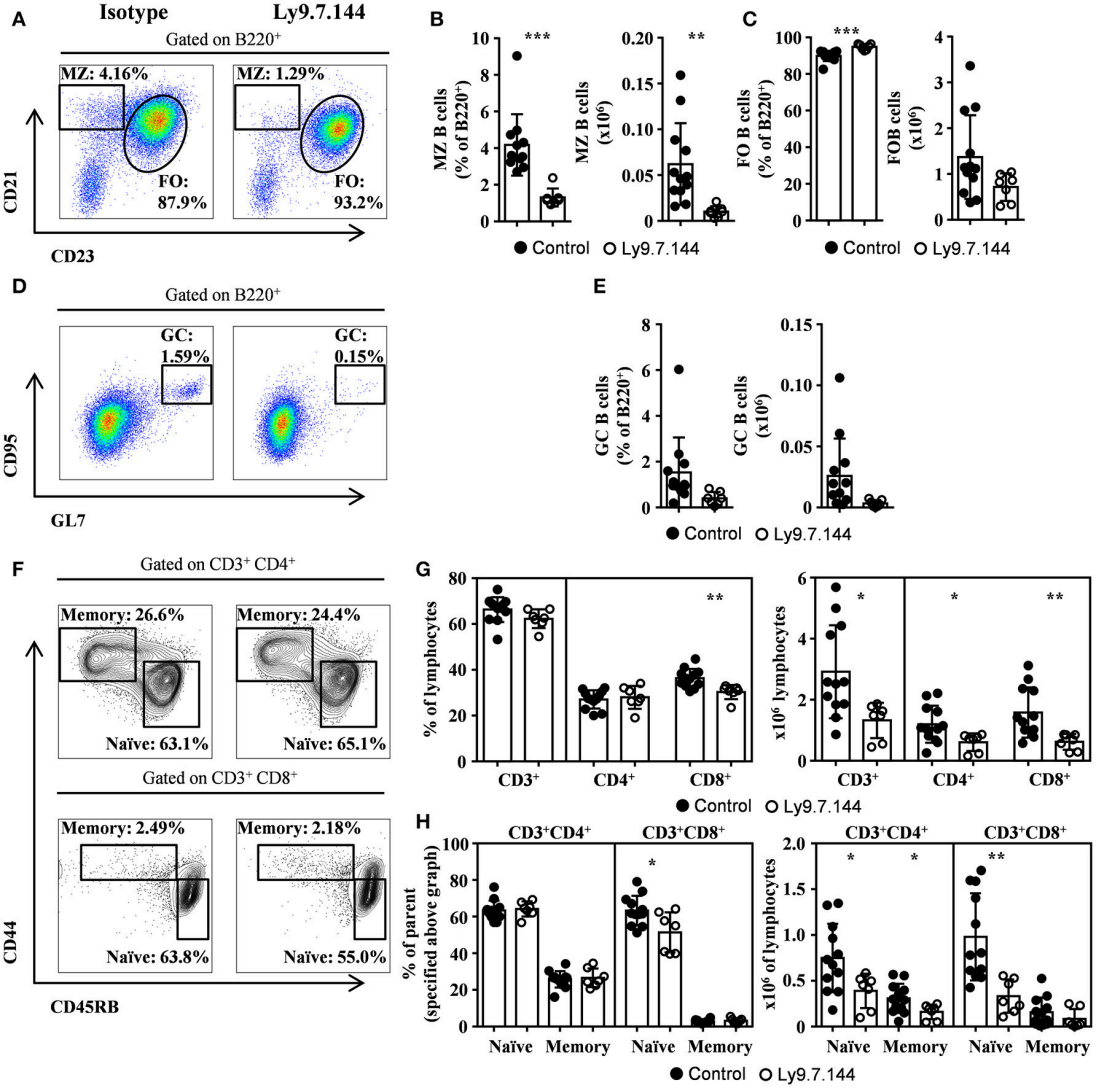
MZ and GC B cells, and naïve T cells are diminished by Ly9.7.144 injection in the draining lymph nodes of SjS mice. Representative dot plots are shown for draining lymph nodes MZ and follicular B cells **(A)**, GC B cells **(D)** and CD4^+^ or CD8^+^ memory and naïve T cells **(F)** from female NOD.H-2^h4^ treated with Ly9.7.144 (right plots) or isotype control (left plots). Percentage and absolute counts for the different cell subsets are shown: MZ B cells **(B)**; follicular B cells **(C)**; GC B cells **(E)**; CD3^+^, CD4^+^, and CD8^+^ cells **(G)** and CD4^+^ or CD8^+^ naïve and memory T cells (**H**; percentages are from parental CD3^+^CD4^+^ or CD3^+^CD8^+^ as specified). Mice treated with Ly9.7.144 mAb are represented as empty dots and mice treated with isotype control are represented as black dots. Results are expressed as mean ± SD. *p < 0.05; **p < 0.01; ***p < 0.001 (unpaired two-tailed t-test). Data are pooled from three independent experiments.

High levels of MZ-like B cells within salivary gland draining lymph nodes were observed (Figures 2A,B). These cells were dramatically reduced after the administration of Ly9.7.144 antibody (Figures 2A,B). On the other hand, follicular B cells were not altered in numbers (Figure 2C). Interestingly, we observed a trend in decreased GC B cells from lymph nodes in percentage and counts (Figures 2D,E; *p* = 0.0723 and 0.0707, respectively). Mice treated with Ly9.7.144 presented a reduction in absolute numbers of lymphocytes (4.69 ± 0.77 × 10^6^ vs. 2.10 ± 0.35 × 10^6^; *p* = 0.0245; Isotype vs. anti-Ly9-treated mice, respectively). Total T cells and, specifically, naïve CD4^+^ and CD8^+^ T cells were also reduced as well as memory CD4^+^ T cells (Figures 2F–H). Thus, anti-Ly9 antibody does not have a dramatic effect on T cells as compared to the B cells in this SjS model.

### Salivary gland of SjS mice show reduced lymphocyte infiltrates after Anti-Ly9 administration

**Figure 3:**
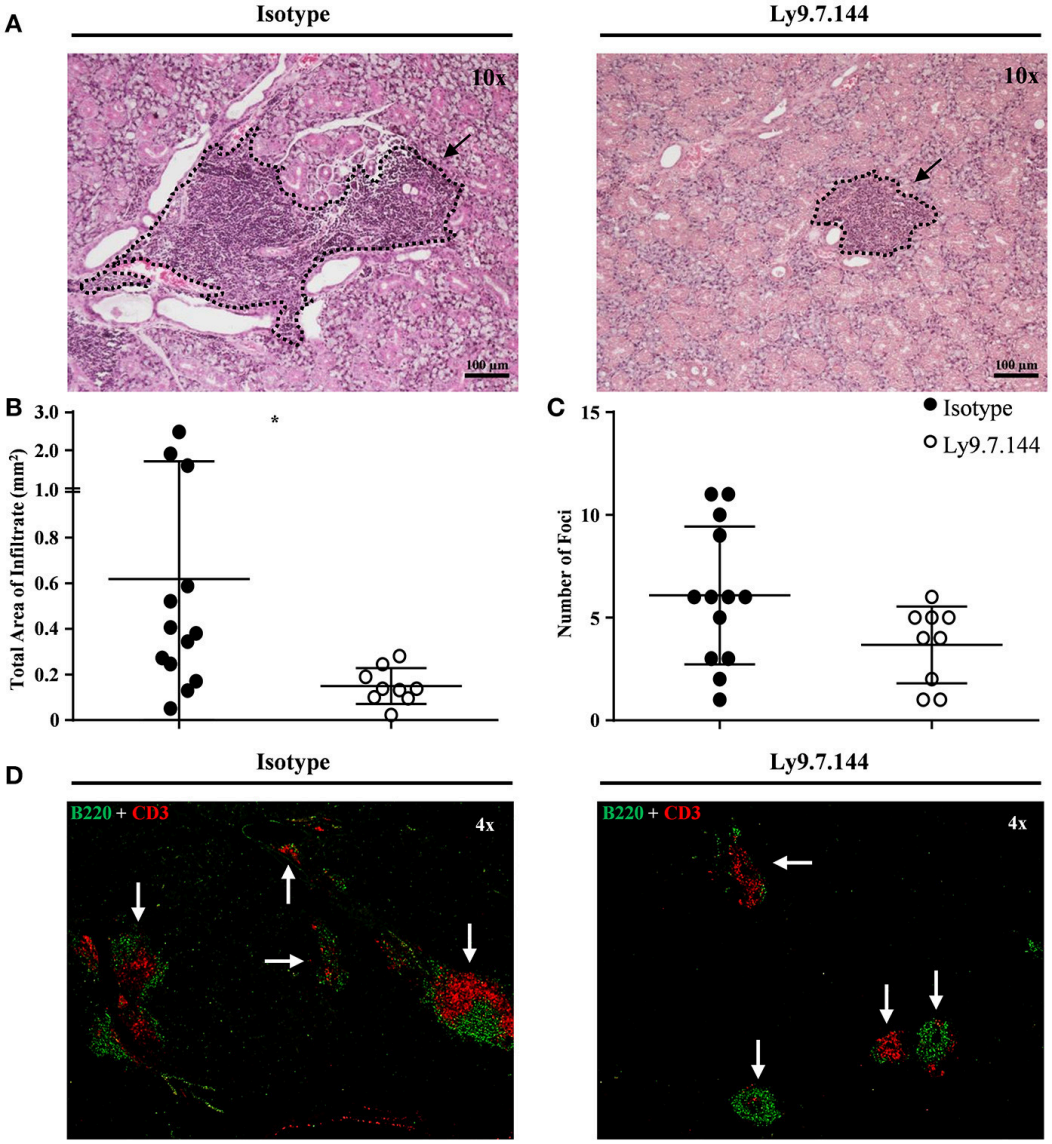
Mice receiving Ly9.7.144 show a reduction in lymphocyte infiltrates area in the inflamed salivary glands of SjS mice. Representative foci from isotype control (left) or anti-Ly9 (right) treated NOD.H-2^h4^ mice found in H&E and immunofluorescence (B220 is shown in green and CD3 in red) staining are shown **(A,D)**. Total area of infiltrating cells **(B)** and foci number **(C)** were calculated from H&E staining of salivary gland paraffin-embedded tissue sections. Three consecutive tissue sections were analyzed per mice to determine foci number. Dotted lines contouring infiltrating areas are indicated with black arrows. The foci was defined as the presence of >50 infiltrating leukocytes in continuum space. Results are expressed as mean ± SD. *p < 0.05 (unpaired two-tailed *t*-test). Data are pooled from four independent experiments.

**Figure 4:**
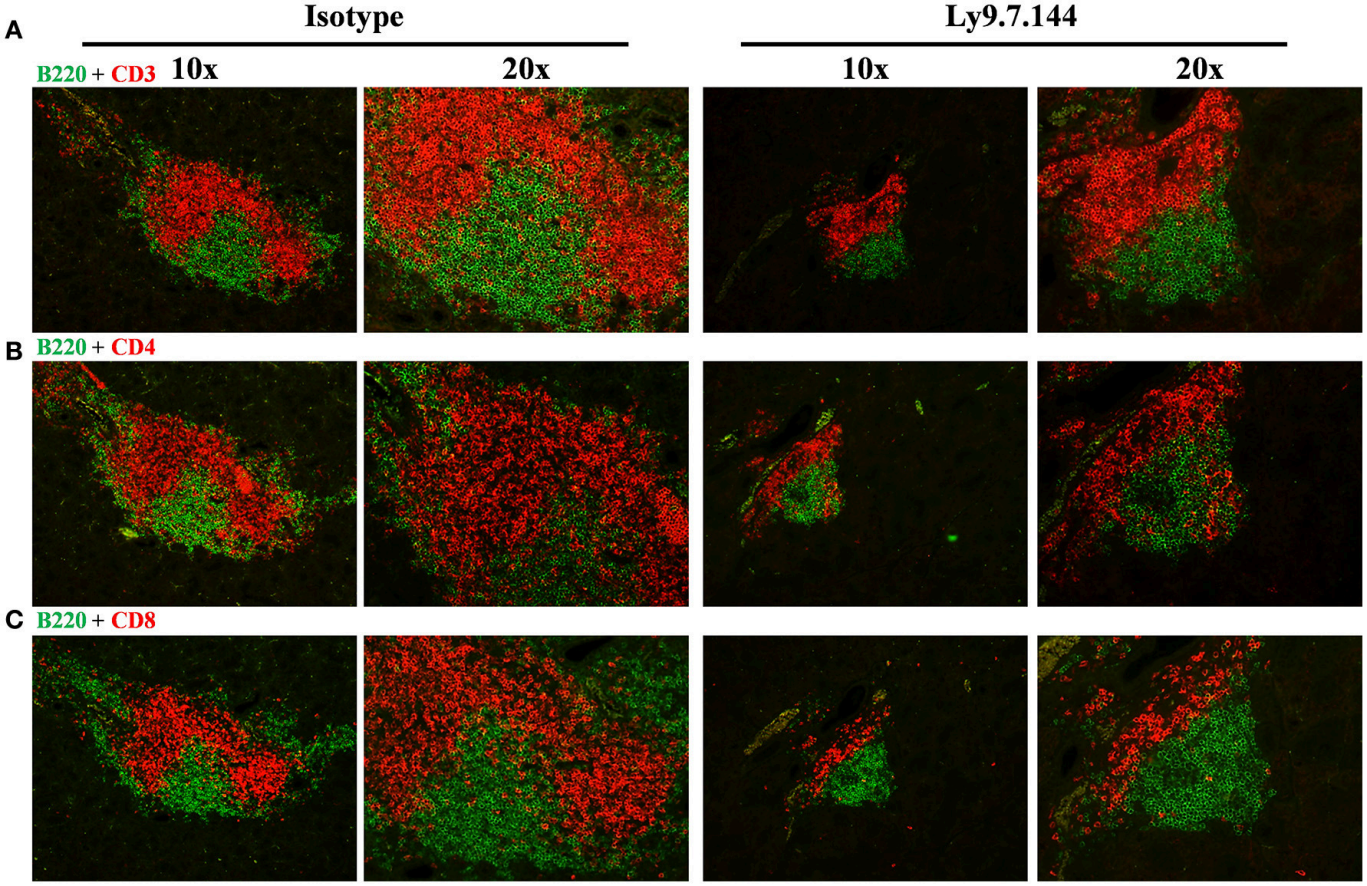
The architecture of salivary gland cell infiltrates is notoriously affected in anti-Ly9-treated SjS mice. Representative immunofluorescence of salivary gland cell infiltrates from anti-Ly9 (right) or isotype control (left) treated NOD.H-2^h4^ mice. The following two color colocalizations at 10 × and 20 × augmentations are shown: B220 [green] + CD3 [red] **(A)**; B220 [green] + CD4 [red] **(B)** and B220 [green] + CD8 [red] **(C)**.

The total area of infiltrating cells in the salivary glands, which is the main target organ of SjS, was significantly decreased in anti-Ly9 treated mice (Figures 3A–D). This reduction was accompanied by a trend reduction of the foci numbers, indicating that the anti-Ly9 treatment was able to reduce the size of this lymphocyte aggregates but not completely eliminate lymphocyte infiltration (Figure 3C). To better study the lymphocyte subpopulations present in the salivary gland infiltrates, we analyzed paraffin-embedded tissue sections by immunofluorescence. Isotype-treated mice showed larger foci formed by T cells surrounded by B cells (Figures 3D, 4A–C). A reduction of both T and B cell infiltrates of mice receiving anti-Ly9 was observed when compared to those mice getting isotype control (Figure 4).

To further characterize the reduction of the B lymphocyte infiltrates of treated mice, we performed a flow cytometric analysis of lymphocytes isolated from collagenase treated tissue. Our results showed a reduction of B cells (CD45^+^B220^+^) and specifically of B cells with a MZ-like phenotype (CD45^+^B220^+^CD21^+^CD23^low^), which are predominant in the inflamed salivary gland (Supplemental Figures 3A–D). Taken together, these results show an important anti-inflammatory effect of anti-Ly9 treatment.

### Ly9 targeting reduces lymphocyte migration into inflamed salivary glands

**Figure 5:**
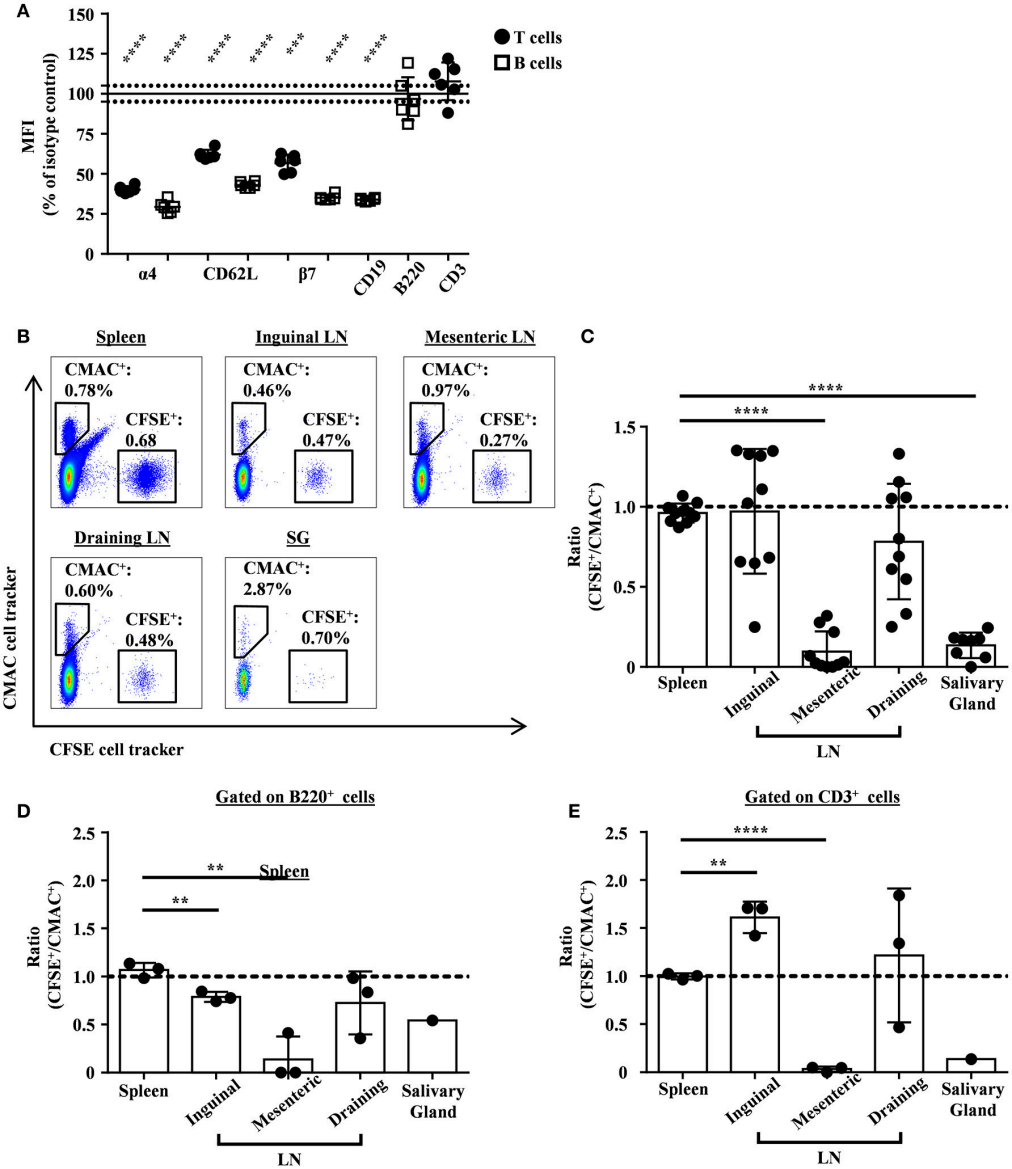
Monoclonal antibody Ly9.7.144 impairs the ability of lymphocytes to migrate into the inflamed salivary glands and mesenteric lymph nodes of SjS mice. Percentages of relative MFI from the spleen of Ly9.7.144-treated mice for 16 h over the mean MFI from isotype-treated mice are shown for the different surface proteins **(A)**. Black dots indicate that MFI values were from pre-gated T cells and empty squares indicate that MFI values were from pre-gated B cells. Results are expressed as mean ± SD. ***p < 0.001; ****p < 0.0001 (unpaired two-tailed *t*-test). Data is from one independent experiment. Representative dot plots for migrating splenocytes into spleen, lymph nodes and salivary gland from age-matched NOD.H-2^h4^ recipient mice are shown **(B)**. Transferred splenocytes from Ly9.7.144-treated NOD.H-2^h4^ mice were stained with CFSE cell tracker. Transferred splenocytes from istoype-treated NOD.H-2^h4^ mice were stained with CMAC cell tracker. Same cell numbers from each mouse were mixed and i.v. administrated. After 24 h the CFSE^+^/CMAC^+^ ratio was calculated for each organ. The ratios of total transferred splenocytes are shown **(C)** as also the ratio of transferred B220^+^ **(D)** and CD3^+^ **(E)** cell subsets. A dashed line indicates ratio value one. Results are expressed as mean ± SD. **p < 0.01; ****p < 0.0001 (unpaired two-tailed *t*-test). Data is pooled from three independent experiments **(C)**. Data is from one independent experiment **(D,E)**. To be able to see the B220^+^ and CD3^+^ transferred cell subsets migrating into the salivary gland, the three salivary glands from the recipient mice were pooled in one unique sample acquisition **(D,E)**.

We previously reported that anti-Ly9 treatment was able to down-regulate several adhesion molecules, including L-selectin (CD62L) and integrin alfa4/beta7, in Balb/c mice (19). Here, we observed that Ly9 targeting also induced an identical effect on NOD.H-2^h4^ mice T and B lymphocytes (Figure 5A). However, we do not exclude that the expression of other lymphocyte adhesion molecules may be affected. To elucidate if the down-modulation of these adhesion molecules could contribute to the observed reduction of the salivary gland lymphocyte infiltration, we performed an *in vivo* two-color lymphocyte migration experiment. We transferred equal numbers of CMAC-labeled splenic lymphocytes from isotype treated mice together with CFSE-labeled splenic lymphocytes from anti-Ly9 treated mice into NOD.H-2^h4^ recipients. After 24 h, the ratio of transferred lymphocytes was analyzed in different tissues. Our results showed that lymphocytes from anti-Ly9 treated mice were much less efficient reaching salivary gland and mesenteric lymph nodes than their isotype treated counterparts, whereas migration into other organs such as spleen, inguinal, and draining lymph nodes was not affected (Figures 5B,C). We could also observe that both B and T cells were equally hindered in their ability to migrate to salivary gland and mesenteric lymph nodes after anti-Ly9 administration (Figures 5D,E). Differences between T and B cells migrating to the inguinal lymph node may be explained by the lower expression of L-selectin on the surface of B cells as compared to T cells.

### Early anti-Ly9 injection prevents onset of salivary gland cell infiltrates

**Figure 6:**
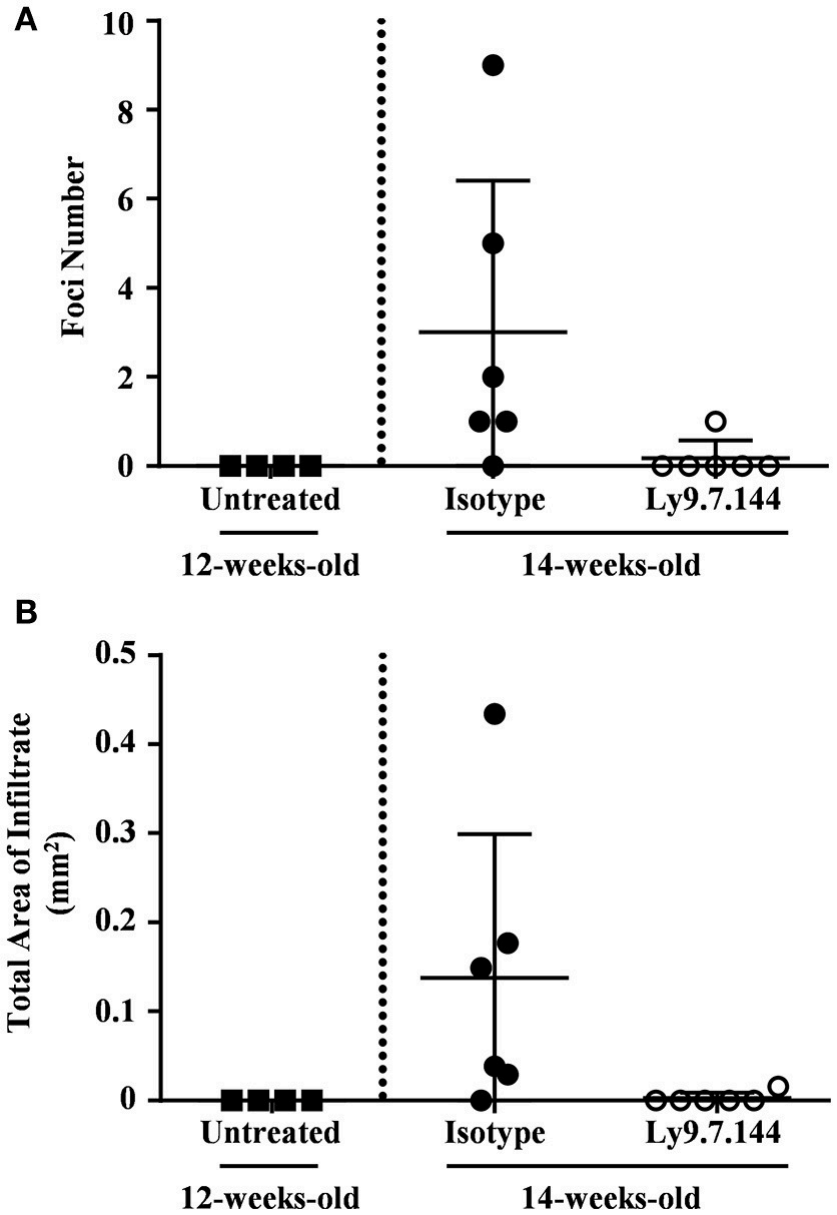
Early administration of anti-Ly9 prevents the apparition of salivary gland cell infiltrates. The foci numbers are compared **(A)** between untreated 12-weeks-old female NOD.H-2^h4^ (black squares) and 14-weeks-old female NOD.H-2^h4^ mice that received a single dose of Ly9.7.144 (empty dots) or isotype control (black dots) for a period of 2 weeks. Same comparison is made with the total area of infiltrate **(B)**. The foci was defined as the presence of >50 infiltrating leukocytes in continuum space. Results are expressed as mean ± SD. (unpaired two-tailed *t*-test). Data is from one independent experiment **(A,B)**.

In order to explore if the ability of Ly9.7.144 mAb to reduce lymphocyte trafficking could affect infiltration into the salivary gland, we treated 12-weeks-old female NOD.H-2^h4^ mice. At this age NOD.H-2^h4^ mice do not present cellular infiltrates (Figures 6A,B). Twelve-week-old NOD.H-2^h4^ mice treated with a single injection of anti-Ly9 for 2 weeks remained without cell infiltrates, whereas most of the mice receiving the isotype control showed several foci and an increased infiltration area (Figures 6A,B). These results strongly suggest that the reduction in lymphocyte migration is a contributing factor to the decline in salivary gland inflammation induced by anti-Ly9 treatment.

### Ly9.7.144 treatment ameliorates kidney inflammation

**Figure 7:**
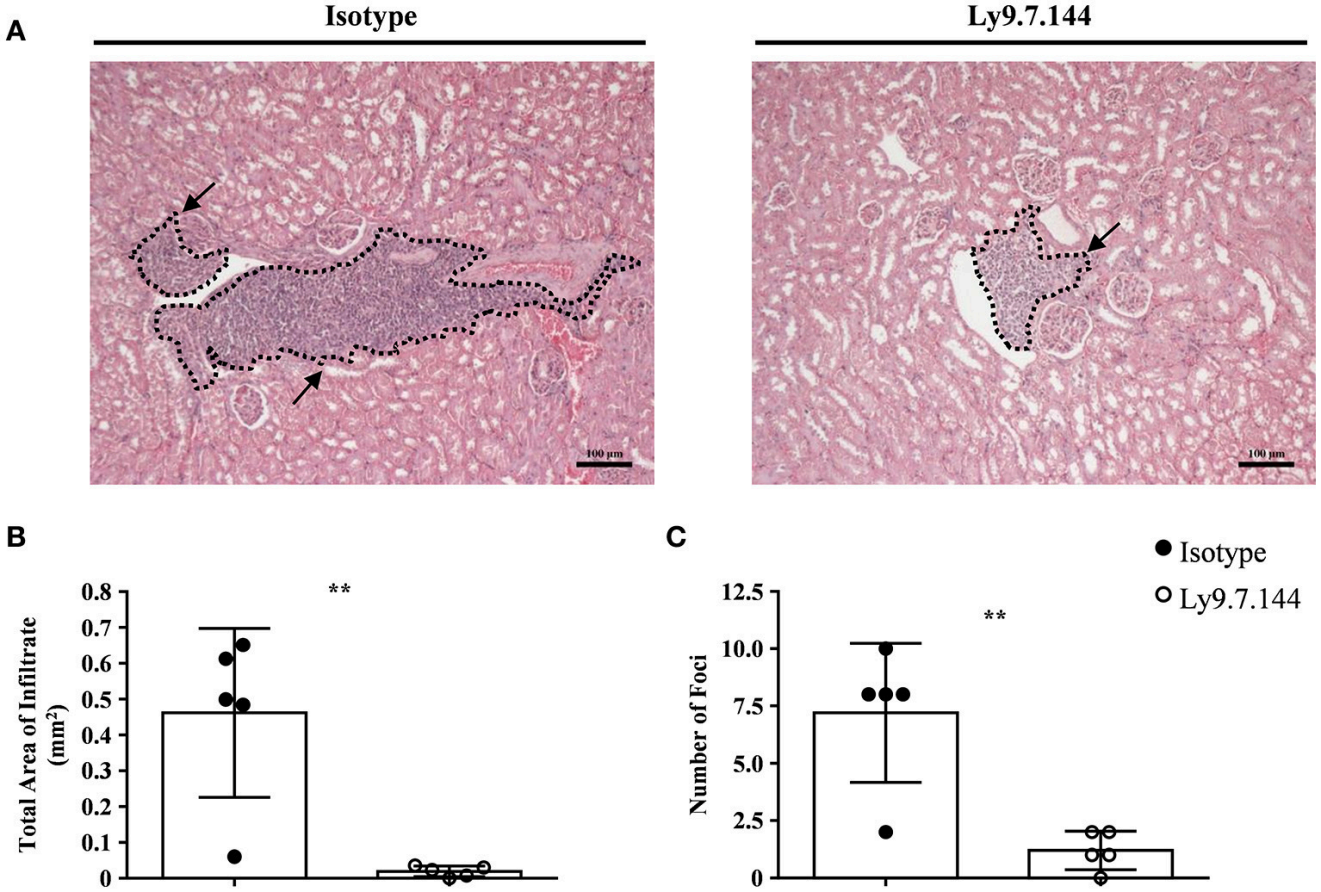
Mice receiving anti-Ly9 treatment show a reduction in renal tubular epithelium infiltrates in SjS mice. Representative foci from anti-Ly9 (right) or isotype control (left) treated mice found in kidney H&E staining are shown **(A)**. Total area of infiltrating cells **(B)** and foci number **(C)** were calculated from H&E staining of kidney paraffin-embedded tissue sections of NOD.H-2^h4^ mice. Mice treated with Ly9.7.144 mAb are represented as empty dots and mice treated with isotype control are represented as black dots. Dotted lines contouring infiltrating areas are indicated with black arrows. The foci was defined as the presence of >50 infiltrating leukocytes in continuum space. Results are expressed as mean ± SD. **p < 0.01 (unpaired two-tailed *t*-test). Data is from one independent experiment **(B,C)**.

Since kidney involvement is a frequent extraglandular manifestation of primary SjS. We next analyzed if mAb Ly9.7.144 could have an effect on kidney inflammation present in NOD.H-2^h4^ mice. To do so, we analyzed paraffin-embedded tissue sections of kidneys from treated mice and compared with those receiving the isotype control. Mice receiving anti-Ly9 mAb presented a dramatic reduction of the total area of cell infiltrates as compared to the control group (Figures 7A,B). Moreover, anti-Ly9 treated mice had significantly fewer numbers of foci (Figure 7C). In contrast, no difference between mice groups in percentage of glomerulosclerosis (obsolete glomeruli per total glomeruli) could be observed (data not shown).

### Ly9.7.144 administration affects autoantibodies production

**Figure 8:**
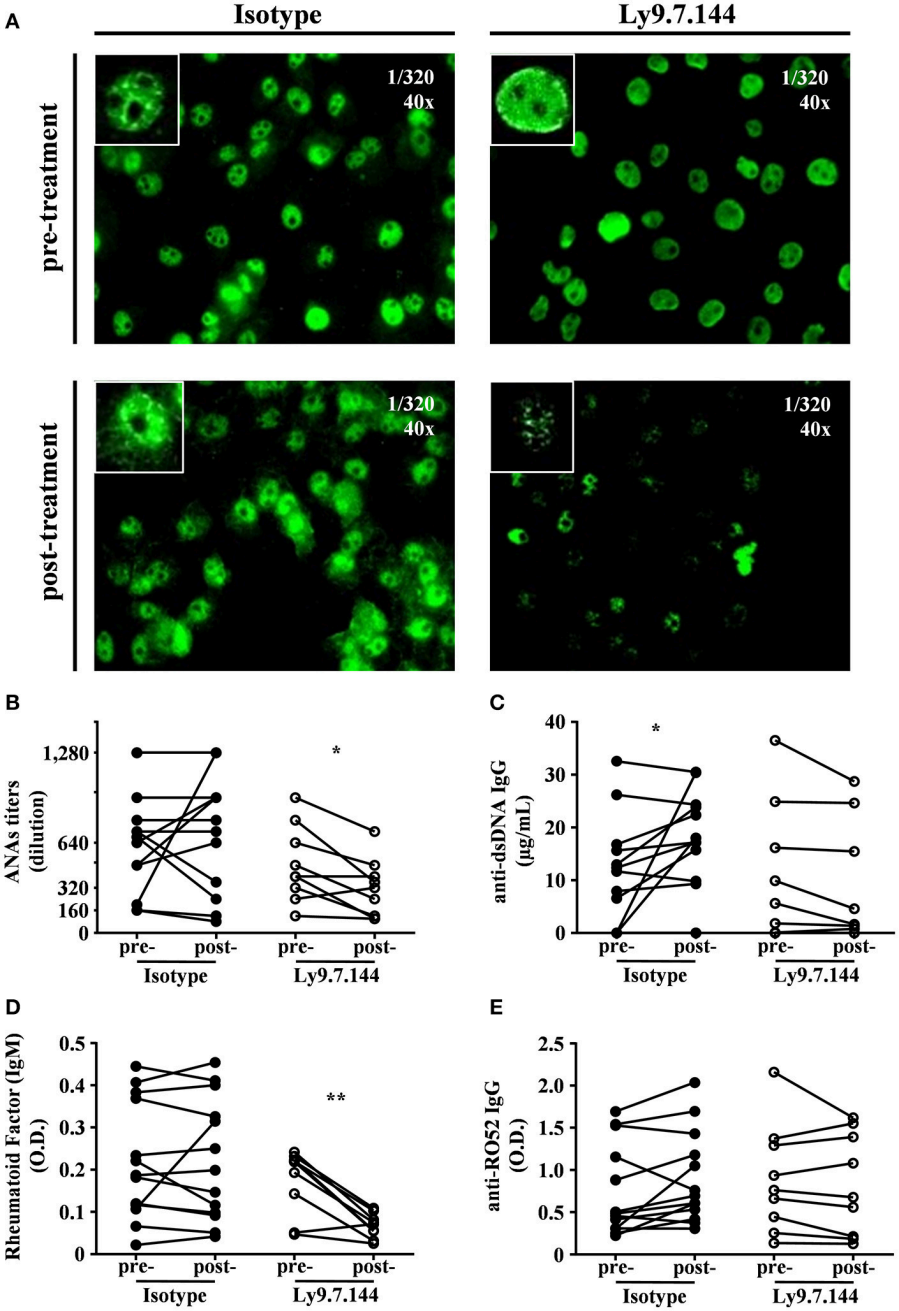
Ly9.7.144 mAb is capable to affect serum autoantibody levels in SjS mice. Photographs of representative ANAs titers **(A)** from pre- (upper panels) and post- (down panels) treatment either with isotype (left panels) or Ly9.7.144 (right panels) mAb are shown. ANAs IgG titers **(B)**, anti-dsDNA IgG **(C)**, rheumatoid factor IgM **(D)**, anti-Ro52 IgG **(E)** autoantibody levels are presented from sera of female NOD.H-2^h4^ mice previous to treatment (24-weeks-old) and after treatment (30-weeks-old). Mice treated with Ly9.7.144 mAb are represented as empty dots and mice treated with isotype control are represented as black dots. To exemplify ANAs the same titer was used at 40 magnifications as specified at the upper right corner of each photograph. Results are expressed as paired values **(B–E)**.*p < 0.05; **p < 0.01 (paired two-tailed *t*-test). Data are pooled from four independent experiments.

We next sought to analyze if anti-Ly9 injection was able to affect anti-nuclear antibodies titers (Figure 8A). A significant decrease in the titers of ANAs was detected in mice receiving Ly9.7.144, whereas no significant change could be observed in the mice injected with the control mAb (Figures 8A,B). Although anti-Ly9 treatment was not able to reduce the levels of anti-dsDNA autoantibodies, these mice did not increase the levels of this autoantibody as observed in the mice treated with the isotype control (Figure 8C). Rheumatoid factor levels were also significantly affected by anti-Ly9 treatment (Figure 8D). In contrast, anti-Ly9 treatment was not able to reduce anti-Ro52 autoantibodies (Figure 8E). These data collectively show that anti-Ly9 is able to have an impact on autoantibody production.

### Treatment with anti-Ly9 induces a slight decrease in plasma cell numbers

**Figure 9:**
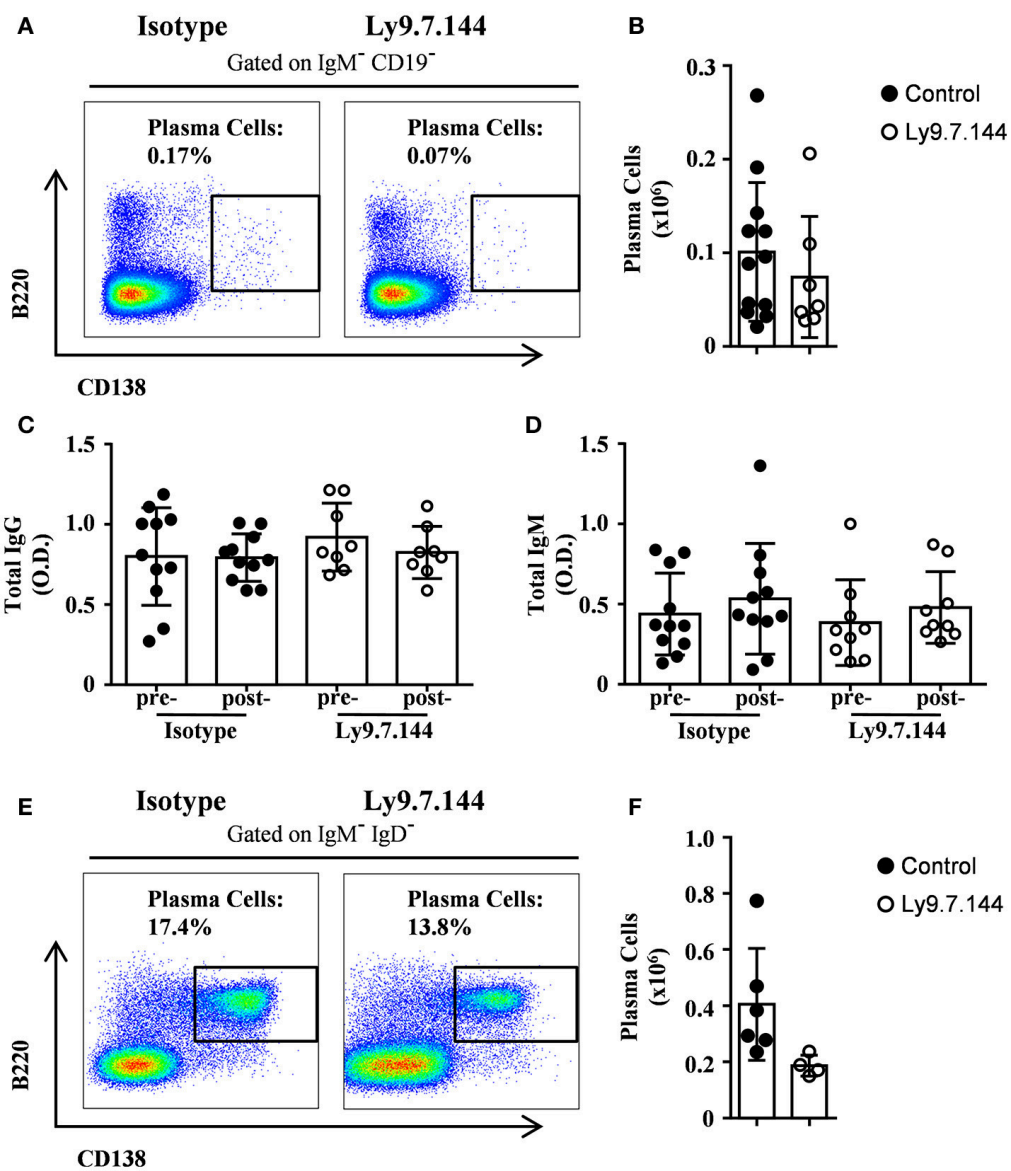
Treatment with anti-Ly9 does not cause general immunosuppression. Representative dot plots of B220 and CD138 are shown for plasma cells from splenocytes gated on CD19^−^IgM^−^ **(A)** or bone marrow cells gated on IgD^−^IgM^−^. **(E)** from 30-weeks-old **(A–D)** or 14-weeks-old **(E,F)** female NOD.H-2^h4^ mice treated with two doses **(A–D)** or a single dose **(E,F)** of Ly9.7.144 (right plots) or isotype control (left plots). Absolute counts for the different plasma cells from spleen **(B)** and bone marrow **(F)** are shown. Total levels of IgG **(C)** or IgM **(D)** are presented from sera of female NOD.H-2^h4^ mice previous to treatment (24-weeks-old) and after treatment (30-weeks-old).Mice treated with Ly9.7.144 mAb are represented as empty dots and mice treated with isotype control are represented as black dots. Results are expressed as mean ± SD. (unpaired two-tailed *t*-test). Data are pooled from three independent experiments **(A–D)** or from one experiment **(E,F)**.

Since Ly9 is also expressed on plasma cells, we analyzed if these cells were affected by anti-Ly9 injection. Splenic plasma cell numbers defined as CD19^−^IgM^−^B220^dim^CD138^hi^ showed a trend to decrease (Figures 9A,B). However, total serum IgG and IgM levels were not disturbed (Figures 9C,D). Finally, we also analyzed the plasma cell numbers in the bone marrow defined as IgD^−^IgM^−^B220^dim^CD138^hi^ after a single injection of Ly9.7.144 for 2 weeks. As with splenic plasma cells, we observed a trend to decrease in numbers (Figures 9E,F). Taken together, these data suggest that anti-Ly9 treatment impairs autoantibody production (Figure 8) without causing general immunosuppression (Figure 9).

## Discussion

The results of this study demonstrate that antibody targeting of the cell surface molecule Ly9 (CD229) can restrain spontaneous autoimmune disease in a mouse model of SjS. We have previously demonstrated that treatment of mice with Ly9.7.144, an agonistic mAb directed against mouse Ly9, had a dramatic effect on splenic MZ and B1 B cells without significantly affecting the number of conventional B cells (19). Since these two subsets of innate-like B cells have been postulated to be essential players in the pathogenesis of SjS in both humans and mice (8, 11, 26–28), we decided to test the hypothesis that injection with this mAb may be able to hinder the development of autoimmunity in NOD.H-2^h4^ mice. Similar to SjS patients, these mice develop spontaneous exocrine gland disease, but in contrast to NOD mice, they do not develop diabetes (20). Moreover, these mice do not only exhibit lymphocytic salivary gland infiltration, but also extraglandular inflammation, such as renal inflammation, mimicking systemic disease manifestations seen in patients (20). In addition, these mice develop autoantibodies to antinuclear antigens such as double-stranded DNA and anti-Ro antibodies, and rheumatoid factor preceding the development of lymphocytic infiltration in the salivary glands (21). In this study, we have injected NOD.H-2^h4^ mice at the age of 24-weeks, when all female mice are known to present salivary gland infiltration, with the mouse anti-mouse Ly9 agonistic mAb, Ly9.7.144. Our results demonstrate that splenic MZ and B1 B cells were diminished after anti-Ly9 mAb injection. In contrast, the number of conventional follicular B cells was found to be increased. MZ and B1 B cells are believed to be responsible for production of autoantibodies to nuclear antigens and other self-antigens, leading to immune complex deposition in tissues (29). It is well known that a high proportion of polyreactive innate-like B cells, such as MZ B cells and B-1 B cells, are reactive with self-antigens. Improper control by immune tolerance mechanisms drives these B cell subsets to produce pathogenic autoantibodies (30). However, experiments with several mouse models of SjS, including NOD.H-2^h4^ mice, indicate that the major pathological function of MZ and B1 B cells is not autoantibody production but T cell activation induced by antigen presentation to T cells and cytokine secretion. MZ B cells have been demonstrated to have the potential to break tolerance to autoantigens by virtue of their capacity to very efficiently present peptides to self-reactive T cells (31). Antigen presentation by B cells is required for development of several spontaneous autoimmune diseases and this is the reason why B cell depletion treatments work in diseases, such as rheumatoid arthritis or multiple sclerosis, where autoimmunity is mainly driven by T cells (32). Interestingly, splenic GC B cells were also significantly decreased in anti-Ly9 treated mice, despite normal or even increased numbers of follicular B cells. We speculate that the lower percentage of GC B-cells is a consequence of the reduced numbers of MZ that are known to carry and present antigens to GC B-cells (33). Thus, it is not surprising that splenectomized and B cell-deficient mice do not develop SjS (20). Moreover, elimination of spontaneous GC in spleens of young NOD.H-2^h4^ mice, by blockade of CD40 ligand, abolished SjS development (34). Therefore, early failure of self-tolerance in the spleen has been proposed to be the initial trigger of the disease. Later in life, this altered response would then be amplified in the gland tissues resulting in disease progression. Importantly, anti-Ly9 injection affected three populations of B cells (MZ, B1 and GC) that have been shown to be more resistant to depletion with CD20 antibodies (35). It is currently unclear if the MZ compartment can be (fully) restored after anti-Ly9 treatment cessation. Determining how anti-Ly9 influences the long-term homeostasis of this subset will be crucial before extrapolating the treatment to SjS patients. Interestingly, the number of T cells was only mildly affected after anti-Ly9. Although we cannot exclude a direct effect of anti-Ly9 administration on the T cells, the decrease in T cell numbers looks reminiscent to that observed after CD20 treatment, indicating that B cells are key for the maintenance of T cell homeostasis (35).

Lymph node MZ-like B cells, also known as nodal MZ, are very infrequent in normal mice but present in autoimmune mice (31). Importantly, these cells are expanded following immunization with autoantigens and can efficiently present these autoantigens to T cells inducing their proliferation (31). Here, we provide evidence that the number of nodal MZ B cells was diminished by anti-Ly9 injection in the draining submandibular lymph nodes probably contributing to the decrease in autoimmune response in the treated mice.

SjS is considered as an autoimmune epithelitis since the lymphocytic infiltrates are found in the epithelial tissues (5). The histological hallmark of both glandular and extraglandular manifestations of SjS is the periepithelial lymphocytic infiltration, characterized by ectopic clusters of T and B cells dominated by CD4 T cells. Chronic inflammation does not only affect epithelial function but also contributes to the autoimmune process by the activation and differentiation of lymphocytes with autoreactive potential within the target tissues (36). Here we demonstrate that anti-Ly9 treatment is able to reduce lymphocyte infiltration in salivary glands. Both T and B cells were reduced in the infiltrated tissue. Our results using a two-color migration assay indicated that anti-Ly9 treated splenic lymphocytes deficiently migrated to the salivary glands. We also observed a dramatic decrease in the migration to the mesenteric lymph nodes without any significant alteration in the migration to the spleen or other lymph nodes. This pattern of altered migration is characteristic of the combined deficiency of L-selectin and integrin beta7 (37). Also, it is well known that alfa4/beta7 integrins are critical for migration of lymphocytes through high endothelial venules associated to mucosa (mesenteric lymph nodes) or tissues with inflammation (38). We speculated that the down-modulation of these two key adhesion molecules by anti-Ly9 affects lymphocyte trafficking, hindering their migration. This could explain the decreased numbers of both T and B lymphocytes in the draining lymph nodes and salivary gland while increasing their accumulation in the spleen. Therefore, targeting of Ly9, by altering the migration of lymphocytes, would prevent the local expansion of autoreactive cells in sites of inflammation in non-lymphoid tissues. Thus, our data point to a dual action of anti-Ly9 treatment, depleting B cells subsets and impacting lymphocyte migration to the inflamed tissues.

Here, we also show that anti-Ly9 treatment was able to significantly reduce renal extraglandular inflammation. Although clinical manifestations of renal failure are observed in around a 10% of SjS patients, recent studies indicate that the prevalence of renal involvement is much higher (39). Renal involvement in primary SjS is the result of epithelial disease with a predominantly lymphocytic infiltration resulting in tubulointerstitial nephritis and glomerulopathy due to the deposition of immune complexes. NOD.H-2^h4^ mice present both kidney alterations, but anti-Ly9 treatment dramatically reduced tubulointerstitial infiltration without a significant impact on altered glomerular structure. This observation is consistent with the rather mild effect of Ly9 targeting on the autoantibody production.

Hypergammaglobulinemia and the presence of autoantibodies are indicators of B cell hyperreactivity in SjS. Here, we observed a reduction of anti-nuclear antibodies and rheumatoid factor. This reduction was very specific since total serum IgG and IgM levels were not affected by anti-Ly9 injection (Figures 9C,D). However, we could not observe a significant inhibition of anti Ro autoantibody production.

In conclusion, our results show that Ly9.7.144 treatment is able to target essential B cells subsets strongly related to the development and maintenance of SjS pathology, and to significantly reduce glandular and extragladular inflammation. In contrast with B cell depletion therapies, such as CD20, the selective deletion of MZ, B1 and GC B cells, with minimal reduction of the conventional B- and T-lymphocyte compartments, should be regarded as an attractive new therapeutic option.

## Author contributions

JP-O performed most of the experiments and analyzed the data. MS and MC performed experiments. AL performed the immunohistology, EC analyzed kidney histology. PE designed the experiments and supervised the study. PE and JP-O wrote the manuscript.

### Conflict of interest statement

The authors declare that the research was conducted in the absence of any commercial or financial relationships that could be construed as a potential conflict of interest.

## References

[1] Brito-Zerón P Baldini C Bootsma H Bowman SJ Jonsson R Mariette X. Sjögren syndrome. Nat Rev Dis Primers (2016) 2:16047. 10.1038/nrdp.2016.47 27383445

[2] Qin B Wang J Yang Z Yang M Ma N Huang F. Epidemiology of primary Sjögren's syndrome: a systematic review and meta-analysis. Ann Rheum Dis. (2015) 74:1983–9. 10.1136/annrheumdis-2014-205375 24938285

[3] Goules AV Tzioufas AG. Primary Sjögren's syndrome: clinical phenotypes, outcome and the development of biomarkers. Immunol Res. (2017) 65:331–44. 10.1007/s12026-016-8844-4 27444892

[4] Ramos-Casals M Brito-Zerón P Font J. The overlap of Sjögren's syndrome with other systemic autoimmune diseases. Semin Arthritis Rheum. (2007) 36:246-255. 10.1016/j.semarthrit.2006.08.007 16996579

[5] Moutsopoulos HM. Sjögren's syndrome: autoimmune epithelitis. Clin Immunol Immunopathol. (1994) 72:162–5. 8050187 10.1006/clin.1994.1123

[6] Wahren-Herlenius M Dörner T. Immunopathogenic mechanisms of systemic autoimmune disease. Lancet (2013) 382:819–31. 10.1016/S0140-6736(13)60954-X 23993191

[7] Kyriakidis NC Kapsogeorgou EK Tzioufas AG A comprehensive review of auto antibodies in primary Sjögren's syndrome: clinical phenotypes and regulatory mechanisms. J Autoimmun. (2014) 51:67–74. 10.1016/j.jaut.2013.11.001 24333103

[8] Shen L Gao C Suresh L Xian Z Song N Chaves LD. Central role for marginal zone B cells in an animal model of Sjogren's syndrome. Clin Immunol. (2016) 168:30–6. 10.1016/j.clim.2016.04.008 27140729 PMC4940264

[9] Kassan SS Thomas TL Moutsopoulos HM Hoover R Kimberly RP Budman DR. Increased risk of lymphoma in sicca syndrome. Ann Intern Med. (1978) 89:888–92. 10.2174/15733955113090100003 102228

[10] Dong L Chen Y Masaki Y Okazaki T Umehara H. Possible mechanisms of lymphoma development in sjögren's syndrome. Curr Immunol Rev. (2013) 9:13–22. 10.2174/1573395511309010003 23853604 PMC3706954

[11] Ambrus JL Suresh L Peck A. Multiple Roles for B-Lymphocytes in Sjogren's Syndrome. J Clin Med. (2016) 5:87. 10.3390/jcm5100087 27740602 PMC5086589

[12] Engel P Eck MJ Terhorst C. The SAP and SLAM families in immune responses and X-linked lymphoproliferative disease. Nat Rev Immunol. (2003) 3:813–21. 10.1038/nri1202 14523387

[13] de la Fuente MA Tovar V Villamor N Zapater N Pizcueta P Campo E Molecular characterization and expression of a novel human leukocyte cell-surface marker homologous to mouse Ly-9. Blood (2001) 97:3513–20. 10.1182/blood.V97.11.3513 11369645

[14] Sintes J Vidal-Laliena M Romero X Tovar V Engel P. Characterization of mouse CD229 (Ly9), a leukocyte cell surface molecule of the CD150 (SLAM) family. Tissue Antigens (2007) 70:355–62. 10.1111/j.1399-0039.2007.00909.x 17919264

[15] Sayós J Martín M Chen A Simarro M Howie D Morra M. Cell surface receptors Ly-9 and CD84 recruit the X-linked lymphoproliferative disease gene product SAP. Blood (2001) 97:3867–874. 10.1182/blood.V97.12.3867 11389028

[16] Li C Iosef C Jia CY Han VK Li SS. Dual functional roles for the X-linked lymphoproliferative syndrome gene product SAP/SH2D1A in signaling through the signaling lymphocyte activation molecule (SLAM) family of immune receptors. J Biol Chem. (2003) 278:3852–9. 10.1074/jbc.M206649200 12458214

[17] Romero X Zapater N Calvo M Kalko SG de la Fuente MA Tovar V. CD229 (Ly9) lymphocyte cell surface receptor interacts homophilically through its N-terminal domain and relocalizes to the immunological synapse. J Immunol. (2005)174:7033–42. 10.4049/jimmunol.174.11.7033 15905546

[18] de Salort J Cuenca M Terhorst C Engel P Romero X. Ly9 (CD229) Cell-surface receptor is crucial for the development of spontaneous autoantibody production to nuclear antigens. Front Immunol. (2013) 4:225. 10.3389/fimmu.2013.00225 23914190 PMC3728625

[19] Cuenca M Romero X Sintes J Terhorst C Engel P. Targeting of Ly9 (CD229) Disrupts marginal zone and B1 B cell homeostasis and antibody responses. J Immunol. (2016) 196:726–37. 10.4049/jimmunol.1501266 26667173 PMC4707085

[20] Braley-Mullen H Yu S. NOD.H-2h4 mice: an important and underutilized animal model of autoimmune thyroiditis and Sjogren's syndrome. Adv Immunol. (2015) 126:1–43. 10.1016/bs.ai.2014.11.001 25727287

[21] Karnell JL Mahmoud TI Herbst R Ettinger R. Discerning the kinetics of autoimmune manifestations in a model of Sjögren's syndrome. Mol Immunol. (2014) 62:277–82. 10.1016/j.molimm.2014.05.006 24907930

[22] Sintes J Cuenca M Romero X Bastos R Terhorst C Angulo A. Cutting edge: Ly9 (CD229), a SLAM family receptor, negatively regulates the development of thymic innate memory-like CD8+ T and invariant NKT cells. J Immunol. (2013) 190:21–6. 10.4049/jimmunol.1202435 23225888 PMC3531811

[23] Cossarizza A Chang HD Radbruch A Akdis M Andrä I Annunziato F. Guidelines for the use of flow cytometry and cell sorting in immunological studies. Eur J Immunol. (2017) 47:1584–797. 10.1002/eji.201646632 29023707 PMC9165548

[24] Yu S Ellis JS Dunn R Kehry MR Braley-Mullen H. Transient depletion of B cells in young mice results in activation of regulatory T cells that inhibit development of autoimmune disease in adults. Int Immunol. (2012) 233–42. 10.1093/intimm/dxs003 22298883 PMC3312073

[25] Stolp J Mariño E Batten M Sierro F Cox SL Grey ST. Intrinsic molecular factors cause aberrant expansion of the splenic marginal zone B cell population in nonobese diabetic mice. J Immunol. (2013) 191:97–109. 10.4049/jimmunol.1203252 23740954

[26] Pers JO Youinou P. Are the B cells cast with the leading part in the Sjogren's syndrome scenario? Oral Dis. (2014) 20:529–37. 10.1111/odi.12153 23837848

[27] Daridon C Pers JO Devauchelle V Martins-Carvalho C Hutin P Pennec YL. Identification of transitional type II B cells in the salivary glands of patients with Sjögren's syndrome. Arthritis Rheum (2006) 54:2280–8. 10.1002/art.21936 16802367

[28] Tsay GJ Zouali M. The interplay between innate-like b cells and other cell types in autoimmunity. Front Immunol. (2018) 9:1064. 10.3389/fimmu.2018.01064 29868023 PMC5964140

[29] Voulgarelis M Tzioufas AG. Pathogenetic mechanisms in the initiation and perpetuation of Sjögren's syndrome. Nat Rev Rheumatol. (2010) 6:529–37. 10.1038/nrrheum.2010.118 20683439

[30] Avrameas S Alexopoulos H Moutsopoulos HM. Natural autoantibodies: an undersugn hero of the immune system and autoimmune disorders-a point of view. Front Immunol. (2018) 9:1320. 10.3389/fimmu.2018.01320 29946320 PMC6005843

[31] Palm AK Friedrich HC Kleinau S. Nodal marginal zone B cells in mice: a novel subset with dormant self-reactivity. Sci Rep. (2016) 6:27687. 10.1038/srep27687 27277419 PMC4899733

[32] Engel P Gómez-Puerta JA Ramos-Casals M Lozano F Bosch X. Therapeutic targeting of B cells for rheumatic autoimmune diseases. Pharmacol Rev. (2011) 63:127–56. 10.1124/pr.109.002006. 21245206

[33] Ferguson AR Youd ME Corley RB. Marginal zone B cells transport and deposit IgM-containing immune complexes onto follicular dendritic cells. Int Immunol. (2004) 16:1411–22. 10.1093/intimm/dxh142 15326094

[34] Mahmoud TI Wang J Karnell JL Wang Q Wang S Naiman B. Autoimmune manifestations in aged mice arise from early-life immune dysregulation. Sci Transl Med. (2016) 8:361ra137. 10.1126/scitranslmed.aag0367 27798262 PMC5291695

[35] Leandro MJ. B-cell subpopulations in humans and their differential susceptibility to depletion with anti-CD20 monoclonal antibodies. Arthritis Res Ther. (2013) 15 (Suppl. 1):S3. 10.1186/ar3908 23566754 PMC3624669

[36] Goules AV Kapsogeorgou EK Tzioufas AG. Insight into pathogenesis of Sjögren's syndrome: Dissection on autoimmune infiltrates and epithelial cells. Clin Immunol. (2017) 182:30–40. 10.1016/j.clim.2017.03.007 28330683

[37] Steeber DA Tang ML Zhang XQ Müller W Wagner N Tedder TF. Efficient lymphocyte migration across high endothelial venules of mouse Peyer's patches requires overlapping expression of L-selectin and beta7 integrin. J Immunol. (1998) 161:6638–47. 9862692

[38] von Andrian UH Mempel TR. Homing and cellular traffic in lymph nodes. Nat. Rev. Immunol. (2003) 3:867–78. 10.1038/nri1222 14668803

[39] François H Mariette X. Renal involvement in primary Sjögren syndrome. Nat Rev Nephrol. (2016) 12:82–93. 10.1038/nrneph.2015.174 26568188

