# Supplementary material for: Ly9 (CD229) Antibody Targeting Depletes Marginal Zone and Germinal Center B Cells in Lymphoid Tissues and Reduces Salivary Gland Inflammation in a Mouse Model of Sjögren's Syndrome
Source: Front Immunol. 2018 Nov 16;9:2661. doi: 10.3389/fimmu.2018.02661 (PMC6251324; doi:10.3389/fimmu.2018.02661)
Supplement: Supplementary file 1 [file Data_Sheet_1.pdf]

A

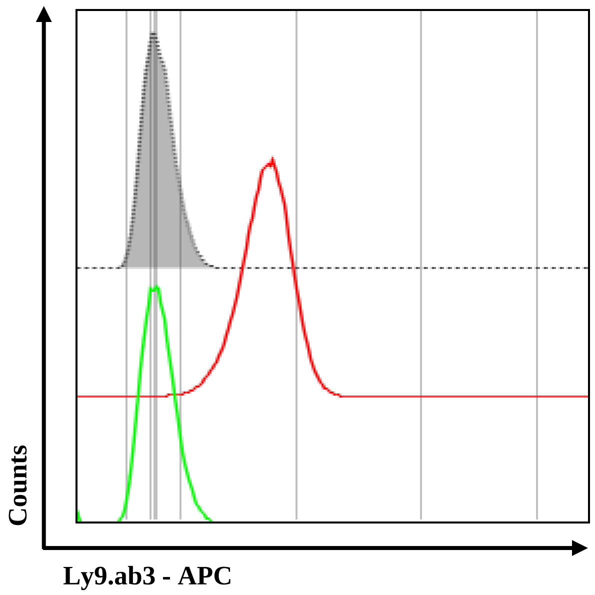


**Supplementary Figure 1. CD229 (Ly9) receptor occupancy.** Representative histograms of Ly9 receptor occupancy are shown (A) for isotype-treated NOD.H-$2^{h4}$ (middle red histogram) and anti-Ly9-treated NOD.H-$2^{h4}$ (down green histogram) mice. Positive signal for Ly9.ab3-APC antibody reveal absence of Ly9.7.144 binding whereas negative signal indicates presence of Ly9.7.144 binding. The grey histogram (upper grey histogram) show the unstained control.

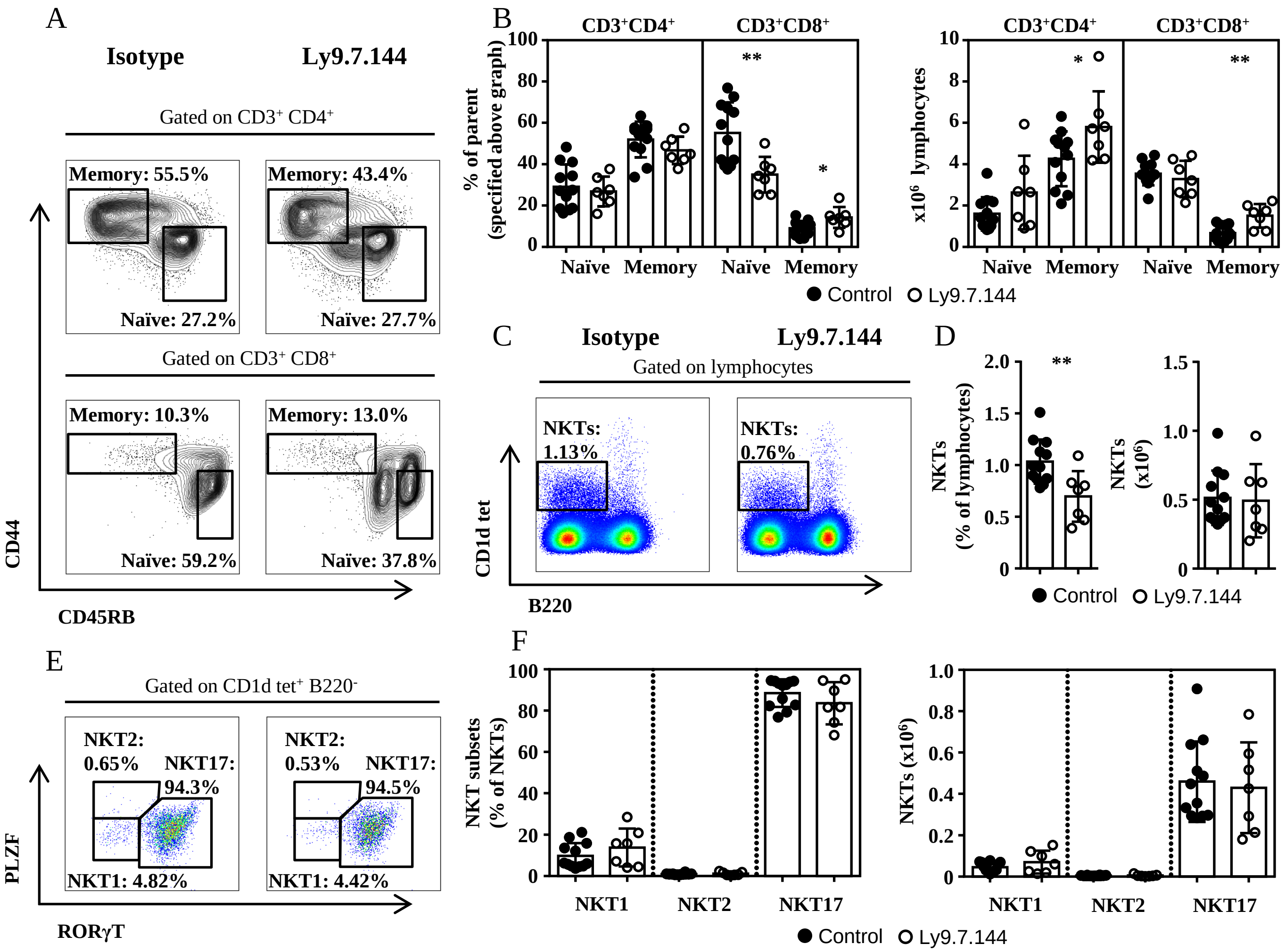


**Supplementary Figure 2. T cell subsets from spleen of anti-Ly9-treated mice are barely affected.** Representative dot plots are shown for splenic $CD4^+$ or $CD8^+$ memory and naïve cells (A); NKT cells (C) and NKT subsets (E) from 30 weeks-old female NOD-$H2^{h4}$ treated with Ly9.7.144 (right plots) or isotype control (left plots). Percentage and absolute counts for the different cell subsets are shown: $CD4^+$ or $CD8^+$ memory and naïve cells [percentages are from parental $CD3^+CD4^+$ or $CD3^+CD8^+$ as specified above graph] (B); NKT cells (D) and NKT cell subsets (F). Mice treated with Ly9.7.144 mAb are represented as empty dots and mice treated with isotype control are represented as black dots. Results are expressed as mean ± SD. *$p<0.05$; **$p<0.01$; ***$p<0.001$ (unpaired two-tailed t-test). Data are pooled from three independent experiments.

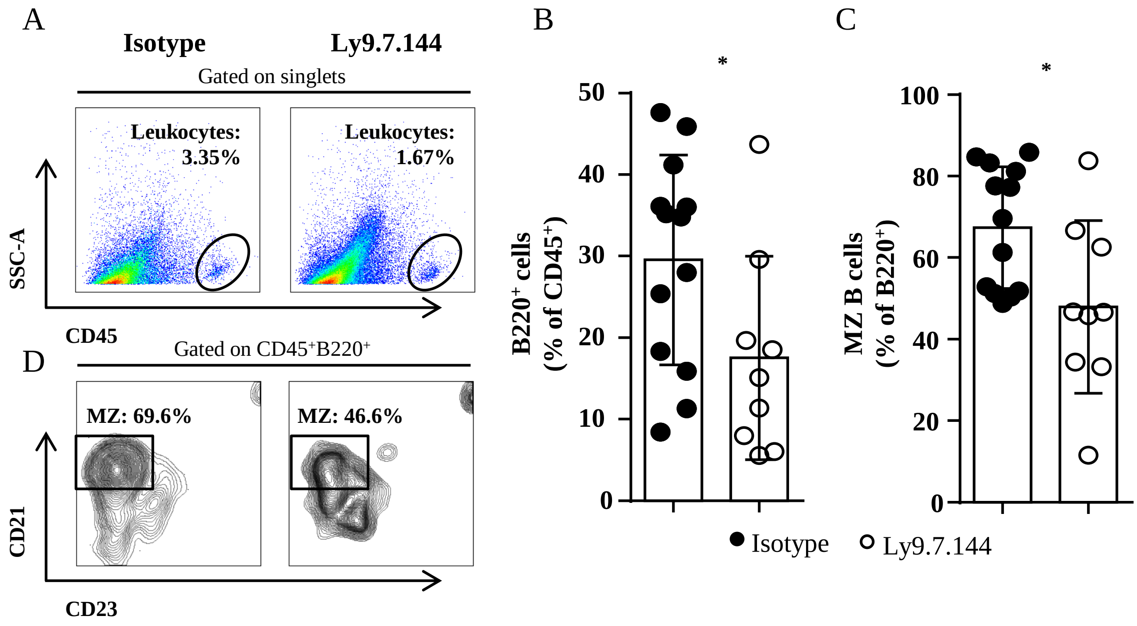


**Supplementary Figure 3. MZ-like B cells infiltrating the inflamed salivary glands are reduced when treated with anti-Ly9.** Representative dot/contour plots are shown for leukocytes (A) and MZ B cells (D) infiltrating the salivary gland from 30 weeks-old female NOD-$H2^{h4}$ treated with Ly9.7.144 (right plots) or isotype control (left plots). Percentages for the salivary gland infiltrating $B220^{+}$ (B) and MZ B cells (C) are shown. Mice treated with Ly9.7.144 mAb are represented as empty dots and mice treated with isotype control are represented as black dots. Results are expressed as mean ± SD. *$p<0.05$ (unpaired two-tailed t-test). Data are pooled from four independent experiments.
